# Effect of transcranial direct current stimulation combined with cognitive rehabilitation on cognitive function and activities of daily living in patients with post-stroke cognitive impairment: a systematic review and meta-analysis

**DOI:** 10.3389/fneur.2025.1523001

**Published:** 2025-06-11

**Authors:** Nan Luo, Binbin Zhao, Hui Wang, Jiabei Wu, Yifan Luo, Man Yuan, Chunlan Xu

**Affiliations:** ^1^Department of Nursing, Beijing Tiantan Hospital, Capital Medical University, Beijing, China; ^2^Nursing College of Shanxi Medical University, Taiyuan, China

**Keywords:** post-stroke cognitive impairment, cognitive rehabilitation, transcranial direct current stimulation, meta-analysis, cognitive function, activities of daily living

## Abstract

**Objective:**

The incidence of post-stroke cognitive impairment (PSCI) has increased alongside the rising prevalence of stroke, making it one of the most serious and prevalent complications among stroke survivors. Growing interest has emerged in whether combined or multi-modal therapies can enhance outcomes through additive or synergistic effects, leading more researchers to investigate the efficacy of transcranial direct current stimulation (tDCS) combined with cognitive rehabilitation (CR) in this population. This study aims to systematically review and meta-analyze the effects of tDCS combined with CR on cognitive function and activities of daily living (ADL) in individuals with PSCI.

**Methods:**

PubMed, Web of Science, Cochrane Library, Embase and China National Knowledge Infrastructure (CNKI) were systematically searched for articles published from inception of the databases through June 2024. Two independent authors screened studies and extracted data. The methodological quality of the included randomized controlled trials (RCTs) was evaluated with the Cochrane Risk of Bias Tool. Meta-analyses were performed using R statistical software (version 4.1.2).

**Results:**

A total of 663 participants across 11 RCTs published between 2013 and 2024 were included. The meta-analysis results indicated that tDCS combined with CR significantly improved cognitive function and ADL among PSCI patients compared to the control group, as evidenced by the Montreal Cognitive Assessment test (MoCA) (MD = 3.03, 95% confidence interval = 2.07 ~ 3.99, *p* < 0.0001), Mini-Mental State Examination (MMSE) (MD = 1.73, 95% confidence interval = −0.05 ~ 3.52, *p* < 0.05), Loewenstein Occupational Therapy Cognitive Assessment (LOTCA) (MD = 11.98, 95% confidence interval = 10.02 ~ 13.93, *p* < 0.0001), Activity of Daily Living Scale (ADLs) (MD = 2.54, 95% confidence interval = 0.76 ~ 4.31, *p* < 0.05), and Modified Barthel Index (MBI) (MD = 5.23, 95% confidence interval = 1.82 ~ 8.64, *p* < 0.01). Subgroup analysis results revealed that tDCS combined with computer-assisted cognitive rehabilitation (CACR) had a greater positive impact on ADL.

**Conclusion:**

tDCS combined with CR significantly improves cognitive function and ADL among individuals with PSCI. Compared with conventional cognitive rehabilitation, the computer-assisted approach demonstrates greater effectiveness in improving ADL among PSCI patients.

**Systematic review registration:**

https://www.crd.york.ac.uk/PROSPERO/, identifier [CRD42024561767].

## Introduction

The Global Burden of Disease (GBD) Study 2021 revealed significant global stroke statistics, 11.9 million new stroke cases, 93.8 million existing cases, and 7.3 million stroke-related deaths worldwide (1). Post-stroke cognitive impairment (PSCI) is a clinical condition characterized by cognitive deficits that emerge within 6 months following a stroke. It is recognized as one of the most prevalent and severe complications of stroke (2, 3). Current epidemiological data show significant variability in PSCI prevalence estimates, with reported rates ranging from 18.0 to 70.0% (4). The long-term functional impairment caused by PSCI often surpasses the initial brain injury, significantly affecting daily activities, rehabilitation progress, and overall prognosis, making it difficult for patients to reintegrate into family and social settings. Furthermore, it heightens the risk of recurrent vascular events, imposing a substantial burden on patients, caregivers, and healthcare systems (5–7).

Current PSCI treatments encompass both pharmacological and non-pharmacological approaches. However, pharmacotherapy often shows limited efficacy and is associated with various adverse effects (8). For example, prolonged use of acetylcholinesterase inhibitors may cause gastrointestinal issues (e.g., diarrhea, constipation), hepatotoxicity, and systemic symptoms such as insomnia and fatigue (9). Consequently, this treatment dilemma has propelled increased research attention toward non-pharmacological interventions such as acupuncture, transcranial magnetic stimulation (TMS), transcranial direct current stimulation (tDCS), and cognitive rehabilitation (CR), which have gained increased scholarly interest (10). Recent studies suggest that tDCS is a more potent non-invasive brain stimulation method compared to TMS (10). It has been shown to alter cortical excitability during stimulation by modulating neuronal resting membrane potentials (11) and to induce neuroplastic effects via prolonged stimulation through glutamatergic synaptic mechanisms (10). tDCS shows significant potential for treating motor dysfunction, mood disorders, and cognitive and speech disorders in stroke patients, owing to its safety, cost-effectiveness, and ease of use (12).

In recent years, tDCS combined with CR has emerged as a focal point of research, with growing interest in whether combined or multi-modal therapies could enhance outcomes through additive or synergistic effects (13–16). Nevertheless, existing research remains controversial regarding whether the combined interventions can achieve the expected effect (17, 18). Currently, the systematic review of tDCS therapy for patients with PSCI has largely overlooked the impact of tDCS combined with CR treatment on these patients (19). Consequently, a comprehensive systematic review and synthesis of the available evidence may be necessary to address this gap and provide clinical staff with the latest evidence-based insights for managing cognitive impairment in stroke patients. This systematic review and meta-analysis aims to evaluate the impact of tDCS combined with CR on both cognitive function and activities of daily living (ADL) in patients with PSCI.

## Materials and methods

This systematic review adhered to the Preferred Reporting Items for Systematic Reviews and Meta-Analyses (PRISMA) guidelines and was registered with the PROSPERO database (CRD42024561767) (20).

### Search strategy

In accordance with the PRISMA Statement, this study conducted a comprehensive literature search to investigate the effects of tDCS combined with CR on the rehabilitation of patients with PSCI. Databases searched included PubMed, Web of Science, Cochrane Library, Embase, and China National Knowledge Infrastructure (CNKI), covering all entries up to June 2024. The search terms included “transcranial direct current stimulation,” “cognitive rehabilitation,” “cognitive rehabilitation training,” “cognitive training,” “stroke,” “post-stroke,” and “cognitive impairment.” These terms were combined using the Boolean operators “OR” and “AND” to construct a comprehensive search strategy.

### Inclusion and exclusion criteria

Eligible studies were required to meet the following inclusion criteria: (1) P (Population) – Stroke patients with cognitive impairment confirmed by cognitive function assessment, where cognitive impairment was caused by stroke rather than other diseases. (2) I (Intervention) – tDCS combined with CR administered in either sequential or synchronized formats. (3) C (Comparison) – tDCS alone; sham tDCS alone; sham tDCS with CR; CR alone; or passive control (i.e., usual care or no treatment). (4) O (Outcomes) – Indicators of cognitive functioning include the Mini-Mental State Examination (MMSE), Montreal Cognitive Assessment (MoCA) and Loewenstein Occupational Therapy Cognitive Assessment (LOTCA), as well as indicators of ADL, which include the Activity of Daily Living Scale (ADLs) and Modified Barthel Index (MBI). (5) S (Study design) – RCTs.

Exclusion criteria included: (1) Publications in languages other than English or Chinese. (2) Interventions involving additional therapies beyond tDCS and CR that could influence cognitive outcomes (e.g., reminiscence). (3) Animal studies, conference abstracts, protocols, quasi-experimental studies, or case reports. (4) Studies with inaccessible full texts or non-public outcome data.

### Study selection and data extraction

Two investigators (LN and YF) independently conducted literature screening in strict accordance with the established inclusion and exclusion criteria. They independently extracted data using a pre-defined checklist that included publication details (year, country, and authors), participant information (sample size, age, and gender), intervention details (characteristics of both the intervention and control groups, frequency of intervention, and duration of follow-up), and outcome measures (cognitive functioning and daily living skills). Any disagreements were resolved through discussion or by involving a third team member (YM).

### Quality assessment

Two researchers (LN and CL) independently assessed the risk of bias in the included studies using the Cochrane “Risk of Bias” tool (21), which evaluates selection, performance, detection, attrition, reporting, and other biases. Each study was rated as having a low, unclear, or high risk of bias. In cases of discrepancies, a third reviewer (JB) was consulted.

### Statistical analysis

The meta-analysis was performed using R statistical software (version 4.1.2). Odds ratios (OR) were used for categorical variables, while mean differences (MD) or standardized mean differences (SMD) were utilized for continuous data. All effect sizes were reported with 95% confidence intervals (95% CI). Heterogeneity among the studies was assessed using the *I^2^* statistic with corresponding *p*-values, where *p* ≥ 0.1 and *I^2^* ≤ 50% indicated no significant heterogeneity, prompting the use of a fixed-effect model. Otherwise, a random-effects model was employed for the analyses. Subgroup analyses were performed as needed to explore potential sources of heterogeneity. When the number of included studies exceeded 10, a funnel plot and Egger’s test were used to assess publication bias. Sensitivity analyses were conducted to evaluate the stability and reliability of the results. *p* < 0.05 was considered statistically significant.

## Result

### Literature search results

**Figure 1:**
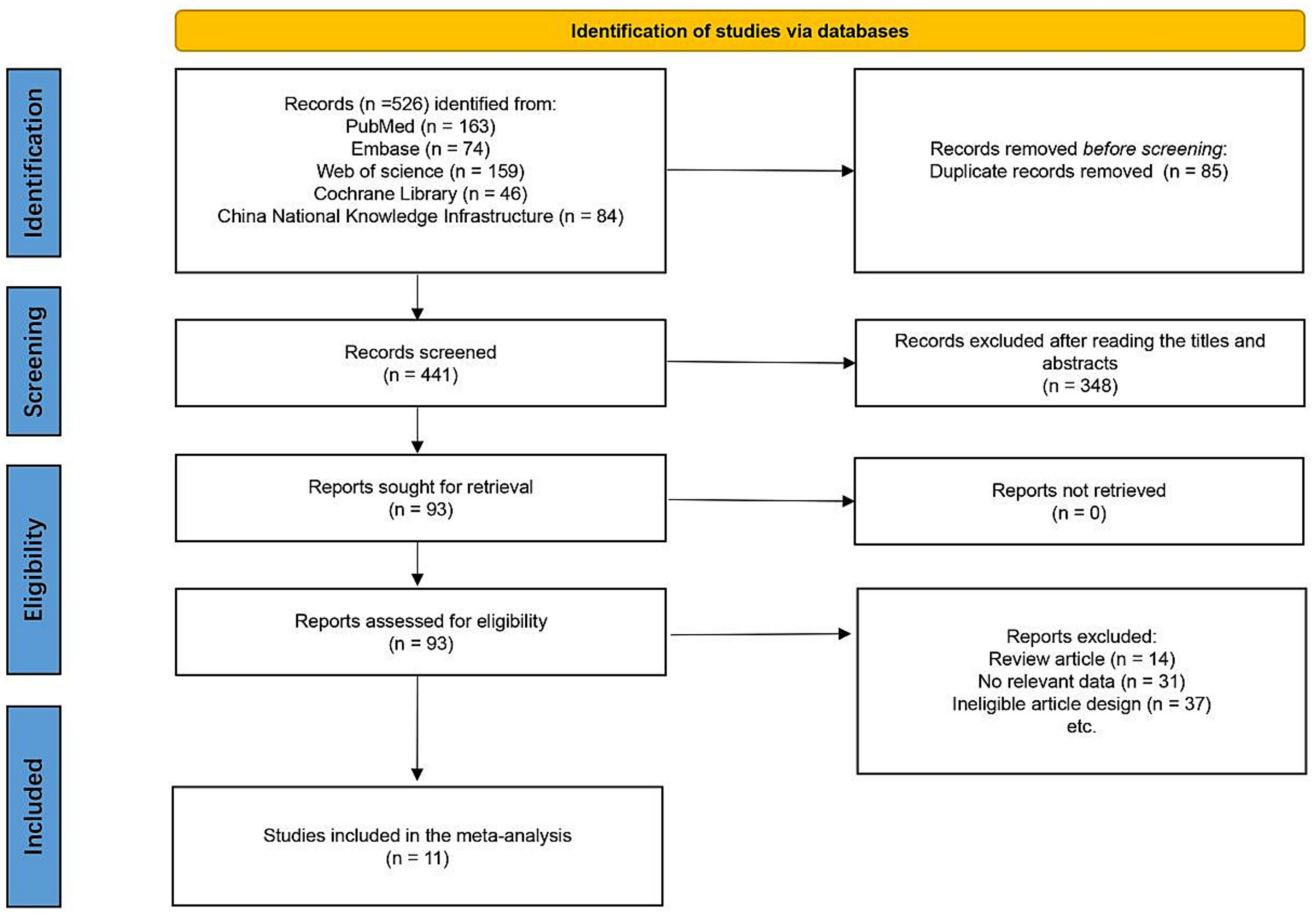
PRISMA flow diagram.

As illustrated in Figure 1, a total of 526 studies were initially retrieved. Among these, 85 were duplicates, and 348 studies were excluded after screening the titles and abstracts. During the full-text review, 82 studies were excluded for not meeting the inclusion criteria, resulting in 11 studies being included in the systematic review (13, 15–18, 22–27).

### The basic characteristics of the included literature

Table 1 summarizes the characteristics of the included studies. The publication years ranged from 2013 to 2024, with over 81.8% of these studies published after 2020. The total sample size of 663 was included, with individual study sample sizes ranging from 11 to 88. Of these, 331 patients were assigned to the experimental group and 332 to the control group. Interventions in the experimental group consisted of tDCS combined with CR or computer-assisted cognitive rehabilitation (CACR). The control group included standalone tDCS, sham tDCS, CR, CACR, sham tDCS combined with CR, sham tDCS combined with CACR, or conventional treatment.

**Table 1:** Characteristics of the 11 included studies.

Study	Intervention		Sample size (*n*)		Age (years)		Gender (M/F)		Type of stroke	Post stroke duration		Target electrode location	Intensity (mA)	Frequency	Treatment duration
	E group	C group	E group	C group	E group	C group	E group	C group		E group	C group				
Park 2013 (17)	tDCS + CACR	sham tDCS	6	5	65.3 ± 14.3	66.0 ± 10.8	2/4	3/2	All stroke	29.0 ± 18.7 days	25.2 ± 17.5 days	Bilateral prefrontal cortex	2	30 min/per time, 5 times/week	18.5 days
Yun 2015 (22)	tDCS + CR (left)	sham tDCS + CR	15	15	60.9 ± 12.9	68.5 ± 14.6	6/9	7/8	All stroke	42.2 ± 31.9 days	39.5 ± 29.6 days	Left fronto-temporal cortex	2	30 min/per time, 5 times/week	3 weeks
	tDCS + CR (right)	sham tDCS + CR	15	15	58.9 ± 15.0	68.5 ± 14.6	7/8	7/8		38.1 ± 27.0 days	39.5 ± 29.6 days	Right fronto-temporal cortex	2	30 min/per time, 5 times/week	3 weeks
Liu 2021 (23)	tDCS + CR	sham tDCS + CR	25	25	65 (60.5, 70.5)	64 (55.0, 70.0)	15/10	12/13	All stroke	8 (7.0, 9.0) months	8 (9.0, 10.5) months	Left DLPFC	2	20 min/per time, 5 times/week	4 weeks
Lv 2021 (24)	tDCS + CR	Conventional treatment	44	44	59.27 ± 10.39	60.44 ± 9.68	19/25	20/24	All stroke	NR	NR	Affected fronto-temporal cortex	1.2 ~ 1.6	20 min/per time, 7 times/week	NR
Ko 2022 (25)	tDCS + CACR	sham tDCS + CACR	12	14	61.25 ± 12.85	57.86 ± 10.04	4/8	8/6	All stroke	≥6 months	≥6 months	Left DLPFC	2	30 min/per time, 5 times/week	4 weeks
Wang 2022 (26)	tDCS + CACR	sham tDCS + CACR	12	12	55.83 ± 14.50	52.33 ± 6.56	7/5	10/2	All stroke	13.33 ± 10.66 weeks	16.00 ± 8.82 weeks	Left DLPFC	2	20 min/per time, 7 times/week	1 weeks
Guo 2022 (13)	tDCS + CACR	sham tDCS + CACR	30	30	55.57 ± 6.26	56.00 ± 5.33	18/12	14/16	Ischemic	11.77 ± 4.34 weeks	11.37 ± 4.69 weeks	Affected DLPFC	2	25 min/per time, 5 times/week	6 weeks
Song 2023 (18)	tDCS + CACR	sham tDCS + CR	18	18	58.94 ± 12.48	59.06 ± 11.15	13/5	12/6	All stroke	23.44 ± 14.11 days	22.62 ± 11.63 days	Affected DLPFC	1.44 ~ 1.98	20 min/per time, 5 times/week	3 weeks
Chen 2024 (16)	tDCS + CACR	tDCS	18	18	61.06 ± 3.08	58.50 ± 3.75	13/5	13/5	Ischemic	22.5 (16.5, 22.5) days	31.0 (24.5, 74.5) days	Left DLPFC	2	20 min/per time, 5 times/week	3 weeks
	tDCS + CACR	CACR	18	18	61.06 ± 3.08	62.44 ± 2.76	13/5	12/6		22.5 (16.5, 22.5) days	38.5 (12.5, 99.3) days	Left DLPFC	2	20 min/per time, 5 times/week	3 weeks
	tDCS + CACR	CR	18	18	61.06 ± 3.08	65.17 ± 3.26	13/5	11/7		22.5 (16.5, 22.5) days	29.5 (15.3, 95.8) days	Left DLPFC	2	20 min/per time, 5 times/week	3 weeks
Zhang 2024 (15)	tDCS + CR	tDCS	30	30	55.70 ± 6.22	58.57 ± 8.02	16/14	14/16	All stroke	1–3 months	1–3 months	DLPFC	2	20 min/per time, 5 times/week	4 weeks
	tDCS + CR	CR	30	30	55.70 ± 6.22	56.50 ± 5.45	16/14	18/12		1–3 months	1–3 months	DLPFC	2	20 min/per time, 5 times/week	4 weeks
Zhou 2024 (27)	tDCS + CACR (together)	sham tDCS + CR (together)	20	20	59.80 ± 9.52	61.20 ± 8.27	9/11	12/8	All stroke	39.87 ± 10.57 days	39.93 ± 11.01 days	Affected DLPFC	2	20 min/per time, 5 times/week	4 weeks
	tDCS + CACR (in turn)	sham tDCS + CR (together)	20	20	59.87 ± 7.84	61.20 ± 8.27	10/10	12/8		40.40 ± 12.36 days	39.93 ± 11.01 days	Affected DLPFC	2	20 min/per time, 5 times/week	4 weeks

E group, experimental group; C group, control group; tDCS, transcranial direct current stimulation; CACR, computer assisted cognitive rehabilitation; CR, cognitive rehabilitation; All stroke, Ischemic and hemorrhage stroke; DLPFC, dorsolateral prefrontal cortex; NR, not reported.

### Quality evaluation of included literature

**Figure 2:**
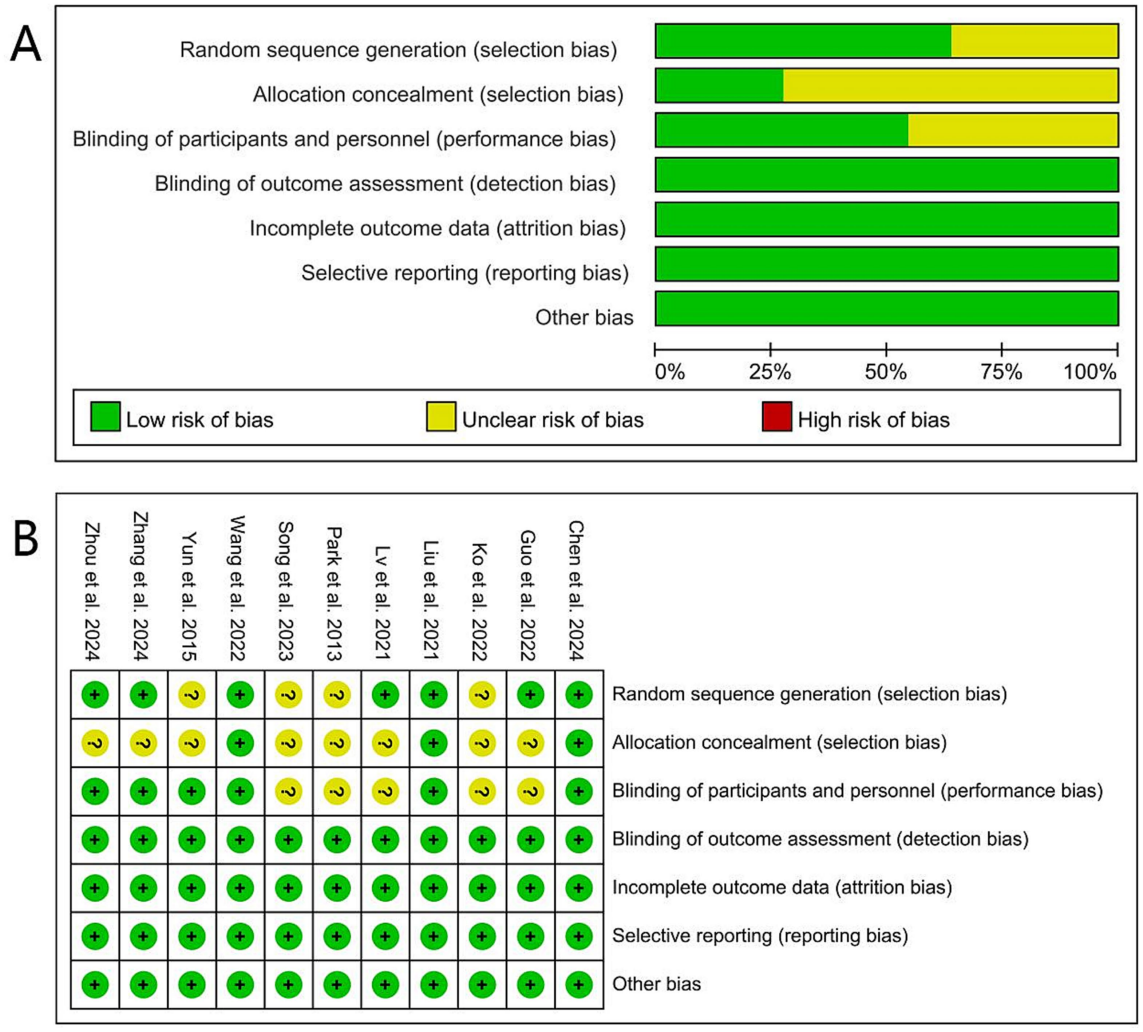
Risk of bias summary and graph: **(A)** The risk of bias profile across. **(B)** The detailed results of the risk of bias.

Figure 2 presents the quality evaluation of the included literature. Out of the 11 randomized controlled trials, three studies were classified as having a low risk of bias, while eight studies had some concerns. Specifically, regarding potential bias in the randomization process, of the 11 studies that were described as randomized, four studies (17, 18, 22, 25) had an uncertain risk due to mentioning randomness without specifying the method, while the remaining seven (13, 15, 16, 23, 24, 26, 27) employed the random number table method, indicating a low risk. For the allocation concealment, three studies (27.27%) (16, 23, 26) showed a low risk of bias, while eight studies (72.73%) (13, 15, 17, 18, 22, 24, 25, 27) did not provide information on whether participants were blinded to group allocation, raising some concerns in this domain. Regarding blinding, six articles (15, 16, 22, 23, 26, 27) demonstrated a low risk, whereas five articles (13, 17, 18, 24, 25) did not mention blinding and were considered to have an uncertain risk. Overall, the quality of the included studies was relatively high.

### Effects of tDCS combined with CR versus control

#### Cognitive function

##### MoCA

**Figure 3:**
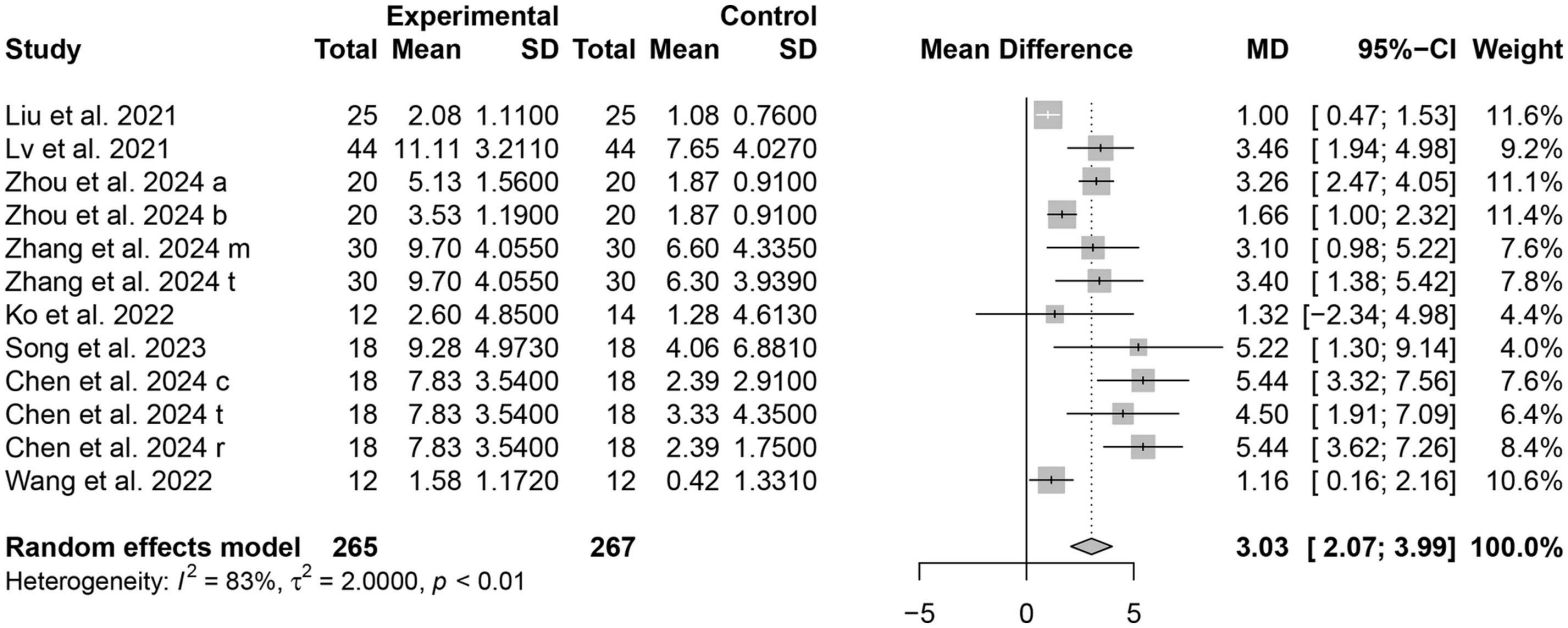
Forest plots of the scores of MoCA.

There are eight studies (15, 16, 18, 23–27) involving a total of 532 patients that assessed the scores of MoCA as an outcome. The results showed that MoCA scores were better in the tDCS combined with the CR group compared to the control group, with a statistically significant difference [MD = 3.03, 95% CI (2.07, 3.99), *I^2^* = 83%, *p* < 0.0001] (Figure 3).

##### MMSE

**Figure 4:**
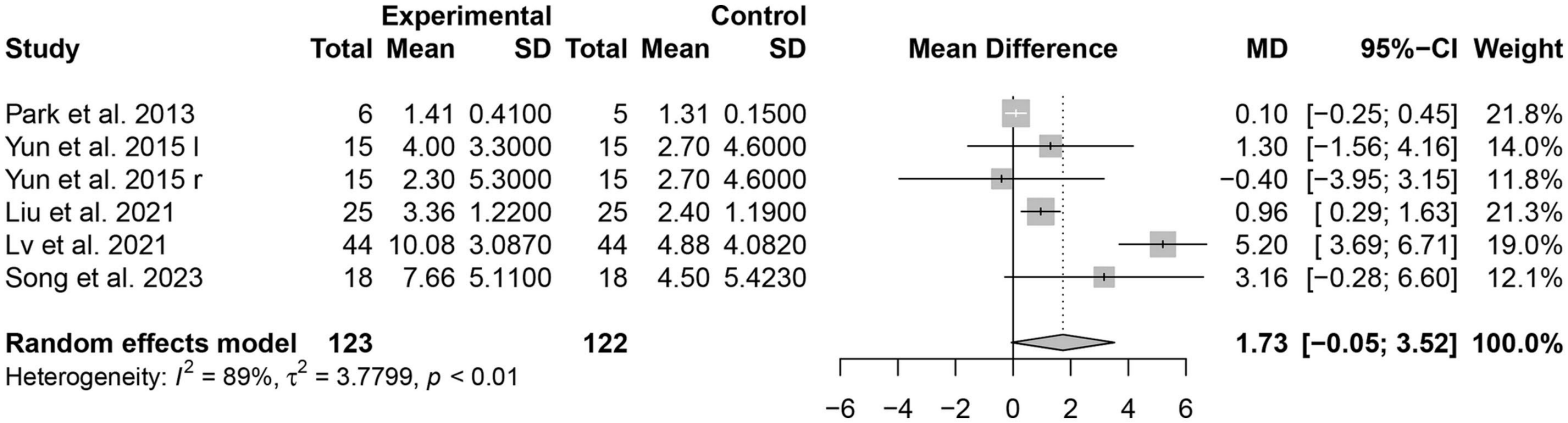
Forest plots of the scores of MMSE.

The effect of tDCS combined with CR intervention on the score of MMSE was evaluated in five studies (17, 18, 22–24) involving 245 patients. tDCS combined with CR demonstrated a significant effect on MMSE scores in patients with PSCI [MD = 1.73, 95% CI (−0.05, 3.52), *I^2^* = 89%, *p* < 0.05] (Figure 4).

##### LOTCA

**Figure 5:**
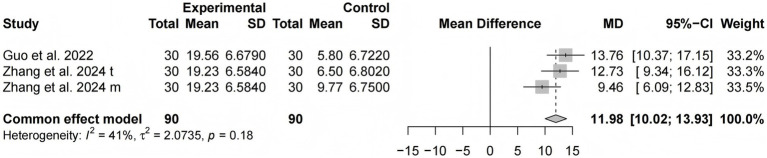
Forest plots of the scores of LOTCA.

Two studies (13, 15) involving 180 participants used the LOTCA to evaluate cognitive function. The results of the meta-analysis revealed that the LOTCA scores in the tDCS combined with the CR group were significantly higher than those of the control group [MD = 11.98, 95% CI (10.02, 13.93), *I^2^* = 41%, *p* < 0.0001] (Figure 5).

#### ADL

##### ADLs

**Figure 6:**
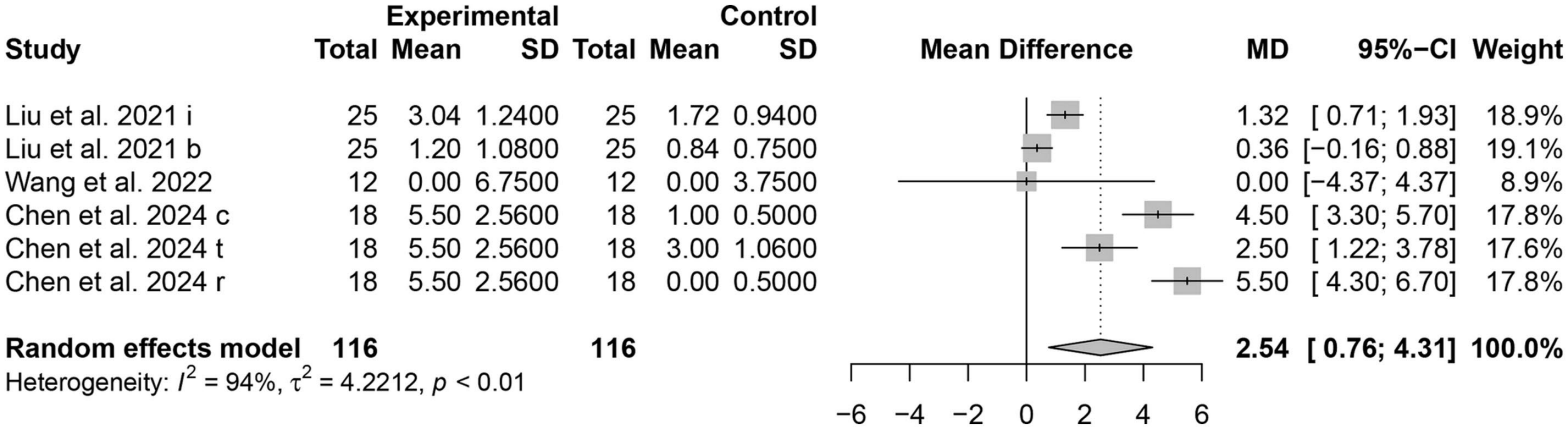
Forest plots of the scores of ADLs.

Three trials (16, 23, 26) reported using ADLs as an outcome index. A total of 232 patients were included, with 116 in the tDCS combined with the CR group and 116 in the control group. The score of ADLs in the tDCS combined with CR group was significantly higher than those in the control group [MD = 2.54, 95% CI (0.76, 4.31), *I^2^* = 94%, *p* < 0.05] (Figure 6). This indicates that the ADL of PSCI patients in the experimental group showed significant improvement.

##### MBI

**Figure 7:**
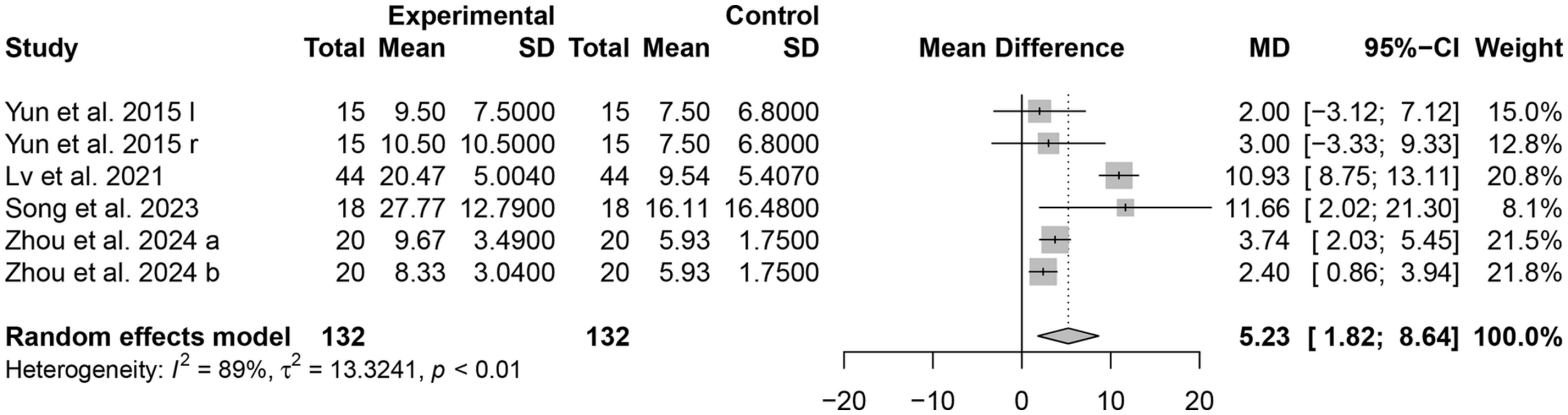
Forest plots of the scores of MBI.

Four studies (18, 22, 24, 27) used the MBI score as an outcome indicator. The heterogeneity test shows that there is significant heterogeneity among the included studies (*I^2^* = 89%, *p* < 0.01), prompting the use of a random-effect model for analysis. This indicates that the efficacy of the experimental group was significantly better than that of the control group, with a statistically significant difference [MD = 5.23, 95% CI (1.82, 8.64), *p* < 0.01] (Figure 7).

### Subgroup analysis

We performed a subgroup analysis of the included studies according to the forms of CR and CACR. The analysis indicated that tDCS combined with CACR was superior to tDCS combined with CR in terms of MoCA, LOTCA, ADLs and MBI scores (Table 2).

**Table 2:** Summary of subgroup analysis results.

Outcome	Studies (*n*)	*I^2^* (%)	Model	MD	*95% CI*	*p*
MoCA	12	83	Random	3.03	(2.07, 3.99)	<0.01
tDCS + CACR	8	82	Random	3.33	(2.00, 4.66)	<0.01
tDCS + CR	4	80	Random	2.53	(1.13, 3.93)	<0.01
MMSE	6	89	Random	1.73	(−0.05, 3.52)	0.06
tDCS + CACR	2	67	Random	1.13	(−1.13, 3.97)	0.43
tDCS + CR	4	89	Random	1.97	(−0.47, 4.41)	0.25
LOTCA	3	41	Common	11.98	(10.02, 13.93)	<0.01
tDCS + CACR	1	–	Common	13.76	(10.37, 17.15)	<0.01
tDCS + CR	2	44	Common	11.09	(8.70, 13.48)	<0.01
ADL	6	94	Random	2.54	(0.76, 4.31)	<0.01
tDCS + CACR	4	80	Random	3.68	(1.82, 5.54)	<0.01
tDCS + CR	2	82	Random	0.83	(−0.01, 1.77)	0.08
MBI	6	89	Random	5.23	(1.82, 8.64)	<0.01
tDCS + CACR	3	54	Random	3.21	(1.79, 4.62)	<0.01
tDCS + CR	3	85	Random	5.81	(−0.20, 11.83)	0.07

### Sensitivity analysis

We removed these studies one by one during the sensitivity analysis to assess the stability and reliability of the merger results. The results of the sensitivity analyses are presented in Supplementary Figures S1–S5. The results indicate that, except for the scores of MMSE, which were influenced by the included literature, the remaining four indicators were not affected by the removal of studies, demonstrating good stability. The stability of MMSE may be related to the small number of studies included.

### Publication bias analysis

**Figure 8:**
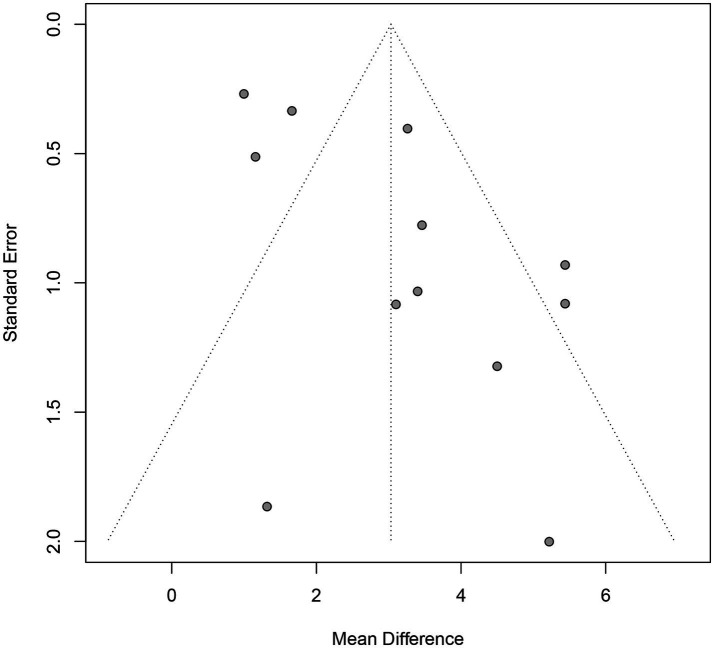
Funnel plot.

Only the MoCA enrollment data in the final variable exceed 10. Publication bias for the included studies was evaluated using the funnel plot and Egger’s test. The distribution of the scatter points is not perfectly symmetrical (Figure 8). Further quantitative analyses of Egger’s test were performed to detect publication bias, which indicated the presence of publication bias (*p* = 0.02).

## Discussion

In recent years, there has been a growing body of literature on tDCS combined with CR to enhance cognitive function in patients. The potential of tDCS combined with CR to improve cognitive function and ADL in patients with PSCI has been demonstrated, although results vary across studies. While a systematic review has evaluated this combination in healthy older adults, none have specifically addressed its impact and effectiveness in PSCI (28). To the best of our knowledge, this represents the first systematic review and meta-analysis to evaluate the synergistic effects of tDCS combined with CR on cognitive function and ADL among patients with PSCI. Our findings indicate that tDCS combined with CR is a relatively effective non-pharmacological intervention that significantly improves cognitive function and ADL in patients with PSCI. This study not only updates the current understanding of the efficacy of combined tDCS and CR but also provides further evidence supporting the advantages of this integrated approach.

This systematic review and meta-analysis analyzed 11 RCTs involving 663 participants. The results indicate that tDCS combined with CR is more effective than CR alone, tDCS alone, or conventional interventions in enhancing cognitive function and ADL in patients with PSCI. As synaptic plasticity is a fundamental property of neurons and is thought to underlie learning and memory (29). The proposed mechanism for the effectiveness of tDCS combined with CR in improving cognitive function in PSCI is that tDCS modulates synaptic plasticity by altering the concentrations of calcium ions and *γ*-aminobutyric acid in astrocytes (30) and the neural basis for the effectiveness of cognitive training lies in brain plasticity (31). Meanwhile, CR, informed by information processing theory, can further guide neuroplasticity and cerebral vasodilation, accelerating recovery in damaged areas of the brain (16, 31). Therefore, tDCS combined with CR suggests the possibility of inducing a positive synergistic effect (27). It aligns with the findings of Chen et al. (16) and Zhang et al. (15). The ADL is influenced by cognitive function, which is also thought to involve higher cognitive processes. In other words, patients’ ADL is also further improved with cognitive functioning (32). Collectively, this suggests that tDCS combined with CR provides an effective neurorehabilitation strategy for patients with PSCI.

We conducted subgroup analyses based on different intervention forms: traditional CR and CACR. We conducted subgroup analyses based on different intervention forms: traditional CR and CACR. The results indicated that tDCS combined with CACR showed greater benefits for ADL. CACR is a form of CR that employs computerized cognitive platforms integrating graphics, audio, video, and virtual reality technologies to enhance memory, attention, executive function, and reaction speed (33, 34). Distinct from traditional CR, CACR offers advantages such as individualization, convenience, entertainment, and objectivity (35, 36). It dynamically adjusts task difficulty levels to match patients’ conditions, ensuring continuity in training. Furthermore, it uses multimedia to stimulate interest and motivation, enhancing engagement and training effectiveness (37). These factors likely underlie the superior ADL improvements observed with CACR.

Among the 11 included trials, only four studies (15–17, 23) documented mild adverse effects associated with tDCS combined with CR, primarily manifested as tingling, skin redness, and itching. However, all were within the patients’ tolerance range and had no significant negative impact. Additionally, no patients were withdrawn from the study due to serious adverse effects based on the results reported in all studies. Thus, it indicates that tDCS combined with CR is a well-tolerated therapeutic approach that is relatively safe for clinical implementation.

Our systematic review and meta-analysis employed a rigorous methodology that included only RCTs, enhancing the comparability of the results. The use of multiple assessment tools for synthesizing the effects of tDCS combined with CR on cognitive function and ADL in PSCI patients improved the accuracy of the study. However, there were some limitations. First, our analysis was restricted to studies in Chinese and English, which may have excluded relevant studies published in other languages that could contain valuable data, resulting in a potential language bias. Second, the current analysis focused on overall cognitive function due to limited data from the included studies, which limits specific evaluations of language, attention, and executive functioning. This may limit our understanding of other specific functions in patients with cognitive impairment. Third, substantial heterogeneity in stimulation parameters (including frequency, intensity, and stimulation sites) and the areas affected by stroke among the included studies limited the feasibility of a more comprehensive subgroup analysis. To address these gaps, future studies should prioritize standardized reporting protocols for stroke lesion characterization. Meanwhile, multi-center RCTs with larger sample sizes are necessary to determine the optimal tDCS and CR protocols, aiming to provide more effective and targeted treatments for PSCI patients. In addition, there is evidence that cognitive function impairment is both long-lasting and dynamic, showing an accelerated decline over 6 years post-stroke (38). This systematic review primarily reflected short-term effects, as the outcomes were measured within one to 6 weeks after the intervention was administered. This limitation underscores the need for long-term follow-up studies examining the effectiveness of tDCS combined with CR on cognitive function and ADL in individuals with PSCI. This will provide more comprehensive and reliable evidence for the efficacy of tDCS combined with CR in treating PSCI.

## Conclusion

This meta-analysis and systematic review suggest that tDCS combined with CR is more effective than tDCS alone, or CR alone, or conventional interventions in improving cognitive function and ADL in patients with PSCI. These findings provide essential guidance for healthcare practitioners on using tDCS combined with CR to support brain health in patients with PSCI, as well as for future empirical research. More comprehensive, high-quality trials with longer follow-up periods are essential for definitive conclusions. In-depth mechanistic studies that integrate brain imaging techniques, humoral biomarkers, and genomic data are also crucial for providing more reliable evidence-based clinical rehabilitation strategies.

## Data Availability

The original contributions presented in the study are included in the article/Supplementary material, further inquiries can be directed to the corresponding author.
