# Supplementary material for: Effect of transcranial direct current stimulation combined with cognitive rehabilitation on cognitive function and activities of daily living in patients with post-stroke cognitive impairment: a systematic review and meta-analysis
Source: Front Neurol. 2025 Jun 11;16:1523001. doi: 10.3389/fneur.2025.1523001 (PMC12188770; doi:10.3389/fneur.2025.1523001)
Supplement: Supplementary file 1 [file Table_1.DOCX]

**
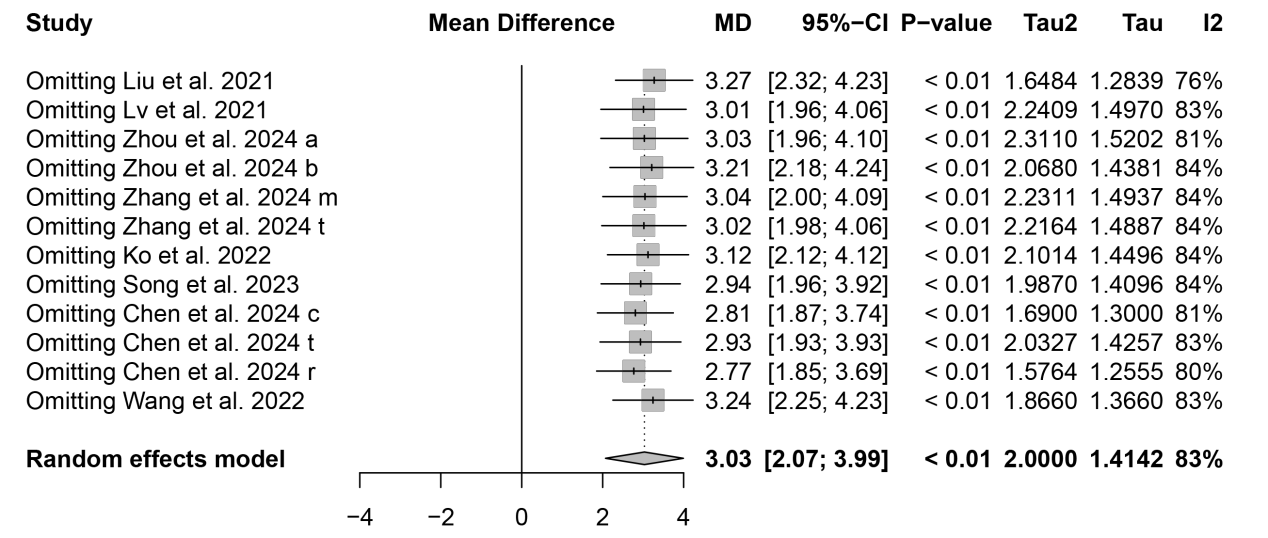
**

**FIGURE S1: Sensitivity analysis of MOCA.**

**
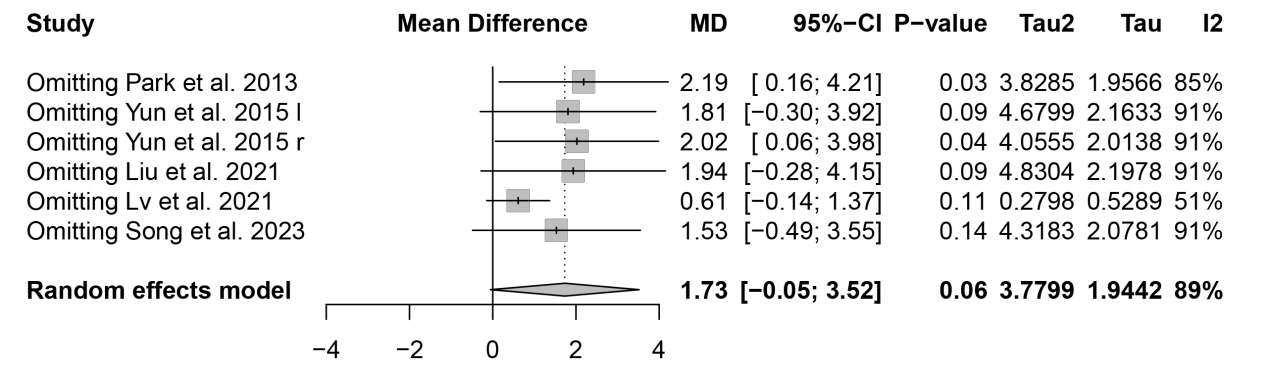
**

**FIGURE S2: Sensitivity analysis of MMSE.**

**
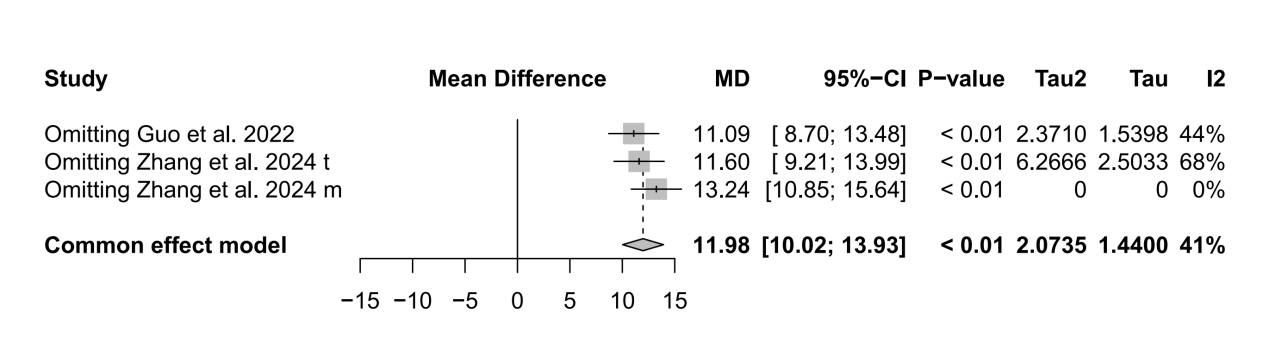
**

**FIGURE S3: Sensitivity analysis of LOTCA.**

**
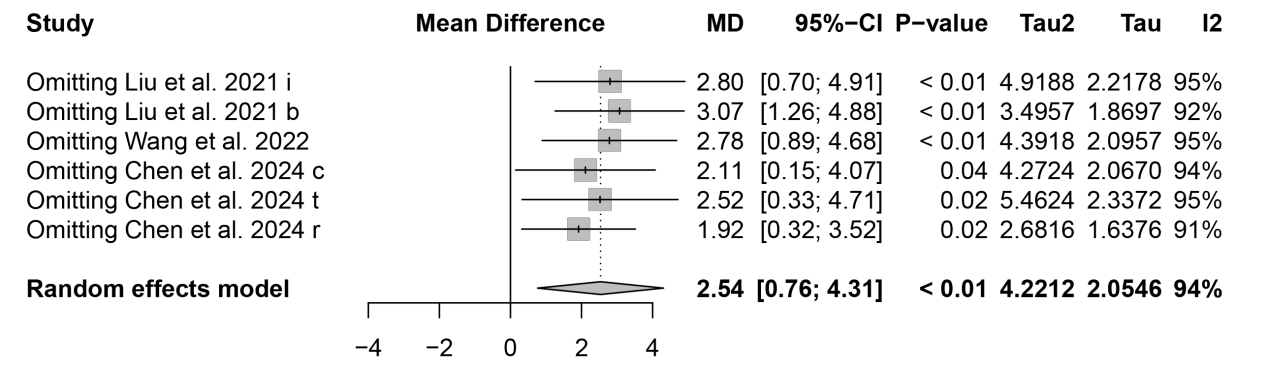
**

**FIGURE S4: Sensitivity analysis of ADLs.**

**
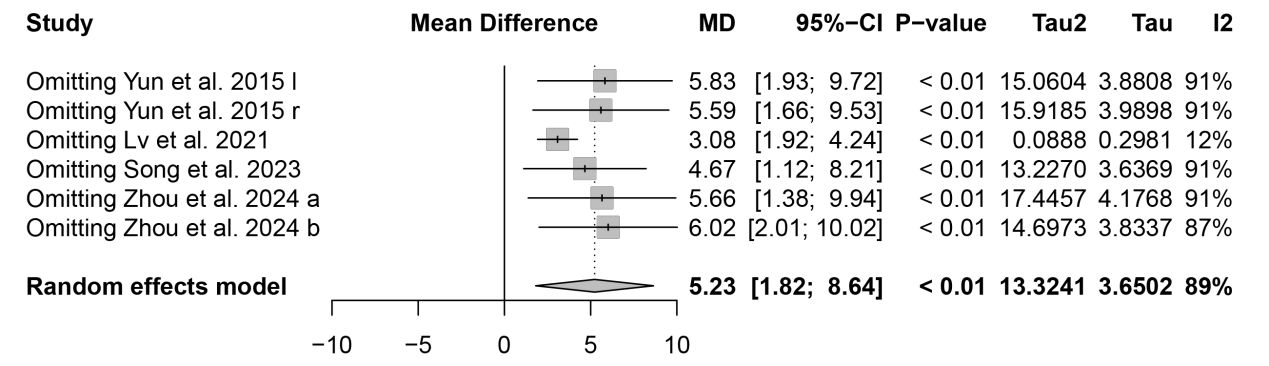
**

**FIGURE S5: Sensitivity analysis of MBI.**
